# Prostate cancer susceptibility gene *HIST1H1A* is a modulator of androgen receptor signaling and epithelial to mesenchymal transition

**DOI:** 10.18632/oncotarget.25536

**Published:** 2018-06-19

**Authors:** Kendra A. Williams, Minnkyong Lee, Jean M. Winter, Derek E. Gildea, Carla Calagua, Natasha L. Curry, Jens Lichtenberg, Huihui Ye, Nigel P.S. Crawford

**Affiliations:** ^1^ Genetics and Molecular Biology Branch, National Human Genome Research Institute, National Institutes of Health, Bethesda, Maryland, USA; ^2^ Computational and Statistical Genomics Branch, National Human Genome Research Institute, National Institutes of Health, Bethesda, Maryland, USA; ^3^ Department of Pathology, Beth Israel Deaconess Medical Center, Harvard Medical School, Boston, Massachusetts, USA; ^4^ Current address: Sanofi, Bridgewater, New Jersey, USA

**Keywords:** ATAC-seq, HIST1H1A, neuroendocrine prostate cancer, epithelial mesenchymal transition, Wnt pathway

## Abstract

In 2018, approximately 165,000 new prostate cancer (PC) cases will be diagnosed, and over 29,000 men will succumb to PC in the U.S. alone. The means of assessing outcome in the clinic are inaccurate, and there is a pressing need to more precisely identify men at risk of aggressive PC. We previously identified *HIST1H1A* as a susceptibility gene for aggressive PC. *HIST1H1A* encodes H1.1, a member of the linker histone family that is involved in chromatin organization and compaction. To understand the molecular basis of aggressive PC, we have characterized how germline variation modulates susceptibility to neuroendocrine differentiation, which is a form of aggressive PC. Immunohistochemistry studies revealed that *HIST1H1A* is over-expressed in normal human prostate tissue compared to prostate adenocarcinoma. Functional characterization of *HIST1H1A* in prostate LNCaP cells indicated that *HIST1HA* over-expression increased cell growth, as well as the expression of neuroendocrine and epithelial-to-mesenchymal markers *in vitro*. Assay for Transposase-Accessible Chromatin (ATAC-seq), which is used to assess chromatin compaction and thus the transcriptional availability of individual genomic regions, demonstrated that H1.1 plays a prominent role in modulating Wnt signaling pathway genes, which are implicated in prostate tumorigenesis. These results demonstrate that *HIST1H1A* is a modulator of aggressive PC susceptibility.

## INTRODUCTION

Prostate cancer (PC) is one of the most commonly diagnosed male cancers in the U.S. It is estimated that approximately 165,000 new PC cases will be diagnosed, and over 29,000 men will die from this disease in 2018 [1]. Measurement of serum prostate specific antigen (PSA) is the established screening tool used for detecting PC. However, there remain legitimate questions regarding the accuracy of this test, since it has no predictive prognostic value at diagnosis which leads to high rates of over-diagnosis and over-treatment [2–4]. Therefore, more in-depth understanding of the mechanisms involved in PC progression is needed to accurately identify men at risk for developing a more aggressive and fatal form of this disease, and to prevent over treatment of men with low risk disease.

Survival and growth of malignant PC cells are dependent on the androgen receptor (AR) signaling pathway. Therefore, androgen deprivation therapy (ADT) with therapeutic agents such as abiraterone and enzalutamide is the first line of treatment for patients suffering from locally advanced PC [3, 5–10]. Many patients exhibit an initial therapeutic response to ADT; however, long term treatment with ADT results in progression to an aggressive, metastatic, and ultimately fatal disease form [11, 12]. Interestingly, autopsy studies have demonstrated that at least 25% of castrate resistant tumors harbor neuroendocrine (NE) histological characteristics, indicating that the incidence of NE prostate cancer (NEPC) is much more common than previously thought [13, 14]. With the widespread use of ADT for treating PC, the incidence of patients with NEPC is expected to rise. Therefore, identifying novel molecular targets, and understanding the mechanisms driving NEPC is of critical importance.

Hereditary variation can contribute considerably to an individual’s risk for developing aggressive and metastatic PC [15–17]. Our earlier study identified aggressive PC susceptibility genes using the C57BL/6-Tg(TRAMP)8247Ng/J (TRAMP) mouse model of NEPC. Quantitative trait locus (QTL) mapping in transgene-positive (TRAMP × NOD/ShiLtJ) F2 intercross males, and expression QTL mapping using primary tumor microarray data identified 35 aggressive PC candidate genes that harbored variants associated with aggressive disease characteristics. Analysis of QTL data demonstrated that differential expression of *Hist1h1a* in prostate tumor samples, as a consequence of germline variation, influences disease aggressiveness in this mouse model. *In silico* analysis identified *HIST1H1A* as having an expression level associated with patient outcome in a human PC gene expression dataset and harboring a single nucleotide polymorphism associated with lymph node metastasis in the PC genome wide association study (GWAS) [15].

The linker histone family member H1.1 forms an integral part of nucleosome, which are the fundamental unit of eukaryotic chromatin. Linker histones interact with both DNA and the core histone octamer to form a unique structural motif that allows for correct folding and compaction of chromatin [18]. Linker histone proteins have several important functions in the nucleosomes. These functions include positioning and spacing within the nucleus, nucleosome stabilization via chromatin compaction, and controlling gene expression by preventing access of transcription factors and RNA polymerase to the DNA [19]. The H1 linker histone family consists of seven somatic variants *H1F0*, *HIST1H1A* (H1.1), *HIST1H1C* (H1.2), *HIST1H1D* (H1.3), *HIST1H1E* (H1.4), *HIST1H1B* (H1.5), and *H1FX* [18]. Interestingly, while the replication-dependent somatic histones H1.2-H1.5 are found depleted in active promoter regions and enriched in areas associated with repression, H1.1 is found enriched in the promoter regions and is associated with transcriptional activity [20]. Additionally, mouse *Hist1h1a* is highly expressed in organs with an abundance of proliferating cells, such as the thymus, spleen, and testis [21].

*In vitro* analyses in our study have relied on two cell lines: LNCaP and PC-3. LNCaP is an AR-positive cell line that is reminiscent of prostate adenocarcinoma. Conversely, PC-3 is an AR-negative cell line that actively expresses NE markers [22] and is thus more comparable to NE prostate carcinoma. In this study, we show for the first time that H1.1 expression is significantly higher in normal human prostate tissue compared to prostate adenocarcinoma. In addition, ectopic expression of *HIST1H1A* suppressed cell growth, invasion and migration *in vitro* in PC-3 cells. Microarray analysis using LNCaP cells indicated that *HIST1H1A* over-expression promotes either an increase or decrease in over 1,900 transcripts. Ingenuity Pathway Analysis (IPA) suggested that both AR signaling and epithelial-to-mesenchymal transition (EMT) pathways are affected in an *HIST1H1A* dependent manner. In line with this finding, protein and differential gene expression data demonstrated that *HIST1H1A* over-expression decreases AR levels and increases EMT markers in an AR positive environment. Assay for Transposase-Accessible Chromatin (ATAC)-sequencing analysis suggested that over-expression of H1.1 impacts the genome landscape in PC cells. Validation of ATAC-seq data using Chromatin Immunoprecipitation and qPCR (ChIP-qPCR), demonstrated that H1.1 occupancy influences important pathways related to aggressive tumorigenesis such as WNT pathway, AR signaling, and EMT. This study is the first to demonstrate a functional role for *HIST1H1A* in influencing aggressive PC susceptibility.

## RESULTS

### *HIST1* gene members are associated with aggressive prostate cancer

**Figure 1:**
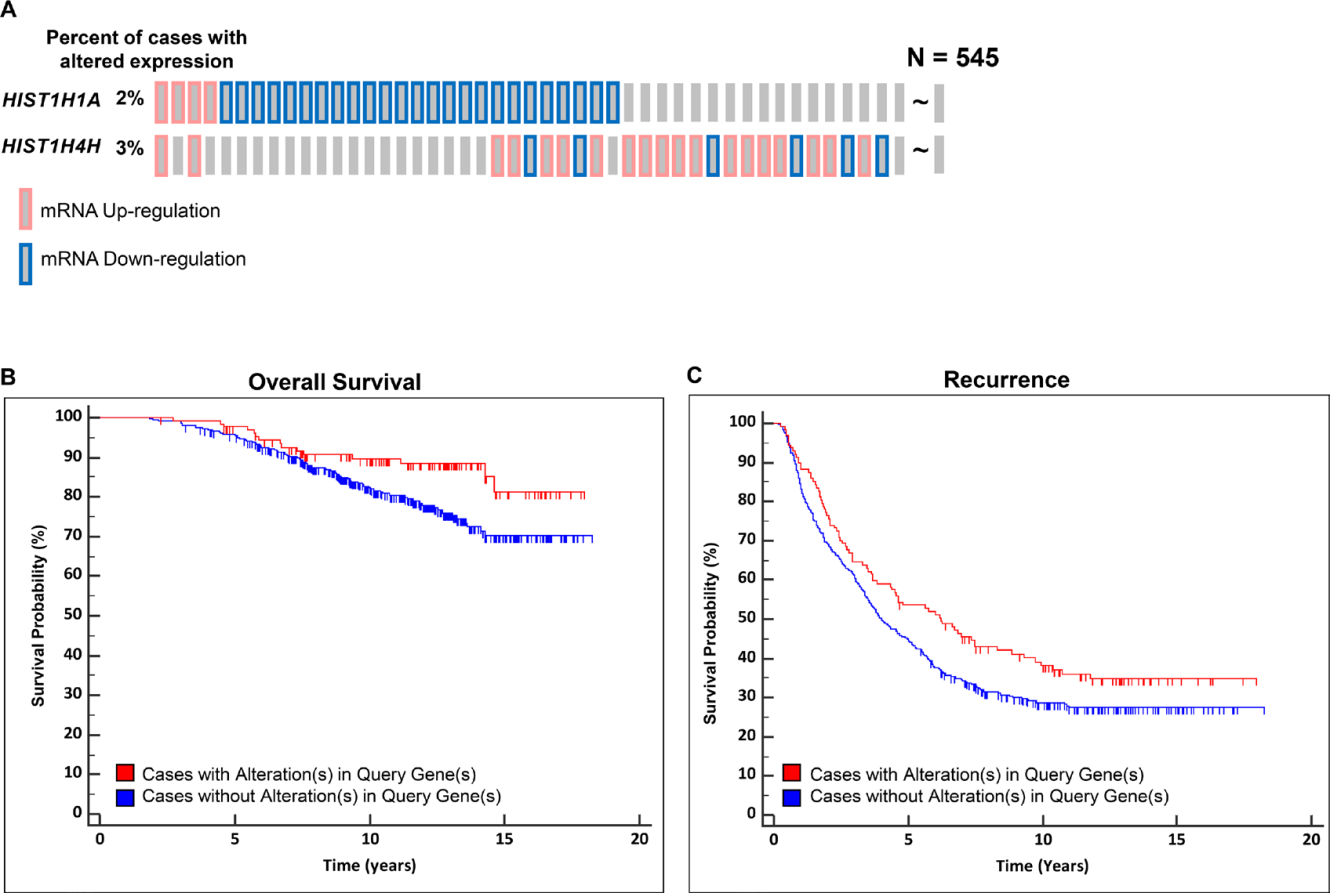
Expression levels of two *HIST1* genes are associated with aggressive prostate cancer outcomes Oncoprint gene expression analysis illustrates the percentage of patients harboring aberrant expression levels of *HIST1H1A* and *HIST1H4H* (**A**). Patients with altered expression of *HIST1H1A* and *HIST1H4H* are associated with better overall survival, log rank *P* = 0.020 (**B**) and reduced disease recurrence, log rank *P* = 0.039 (**C**).

A systems genetics approach in (TRAMP x NOD/ShiLtJ) F2 intercross males was previously used to identify 35 aggressive PC modifier genes. Of these genes, six *Hist1* family members were identified as being associated with susceptibility to aggressive PC (*Hist1h1a, Hist1h2ab, Hist1h3c, Hist1h3e, Hist1h4a,* and *Hist1h4h*) [15]. In this study, further analysis determined that the peak regions of linkage of two loci on mouse Chr. 13 associated with primary tumor burden and nodal metastasis burden were in proximity to the mouse *Hist1* locus. This is of interest since an earlier family-based linkage study demonstrated that the syntenic region of the human genome encompassing the *HIST1* locus (Chr. 6p22.3) is a risk locus for aggressive PC [23]. Given the prominence of *Hist1* locus genes in the list of 35 aggressive PC susceptibility candidate genes in (TRAMP × NOD/ShiLtJ) F2 males, we analyzed the relationship between expression levels of the human orthologs of the six *Hist1* genes and aggressive PC. An *in silico* validation using logistic regression (LR) analysis to determine the correlation between the expression level of all six *HIST1* genes and aggressive PC clinical variables was performed using human PC gene expression datasets. LR analysis was performed using three different cohorts: the Cancer Genome Atlas [TCGA] prostate adenocarcinoma [PRAD] (*n* = 497) [24]; GSE21032 (*n* = 150) [24]; and GSE49961 (*n* = 545) [25] which consist of microarray datasets. These analyses indicated that of the six *HIST1* genes identified in (TRAMP × NOD/ShiLtJ) F2 intercross males, the expression levels of *HIST1H1A* and *HIST1H4H* were associated with aggressive PC clinical variables. In the GSE21032 cohort, *HIST1H1A* expression was associated with Gleason Score (odds ratio = 0.39; 95% CI = 0.22 – 0.67; *P* = 8.00 × 10^–4^; FDR = 0.03). In the TCGA cohort, *HIST1H4H* expression was associated with nodal stage (odds ratio = 1.63; 95% CI = 1.26 – 2.11; *P* = 2.00 × 10^–4^; FDR = 0.01). Additional results for all six *HIST1* genes are shown in Supplementary Table 1. To further examine the association of *HIST1H1A* and *HIST1H4H* expression with survival, we performed Kaplan–Meier survival analysis. The analyses revealed that subjects exhibiting differential expression of either of these genes in primary tumors in the GSE46691 cohort exhibited an improved overall survival and a lower risk of disease recurrence. Specifically, the expression of *HIST1H1A* and *HIST1H4H* were significantly altered in 9.9% (54/545) of the cases (Figure 1A and Supplementary Table 2). Both overall survival (Figure 1B) and disease recurrence (Figure 1C) were significantly improved in patients with higher than average gene expression of *HIST1H1A* and lower than average gene expression of *HIST1H4H* in primary tumors compared to patients with apparently normal levels of these two genes (log-rank *P* = 0.020 and 0.039 respectively), indicating that higher than average gene expression of *HIST1H1A* and lower than average gene expression of *HIST1H4H* were associated with a lower likelihood of aggressive disease. No association between *HIST1H1A* and *HIST1H4H* expression and survival was observed in the GSE21032 and TCGA cohorts (data not shown).

### Characterization of *HIST1H1A* in prostate tissue microarray reveals higher expression in prostate normal tissue compared to prostate adenocarcinoma

**Figure 2:**
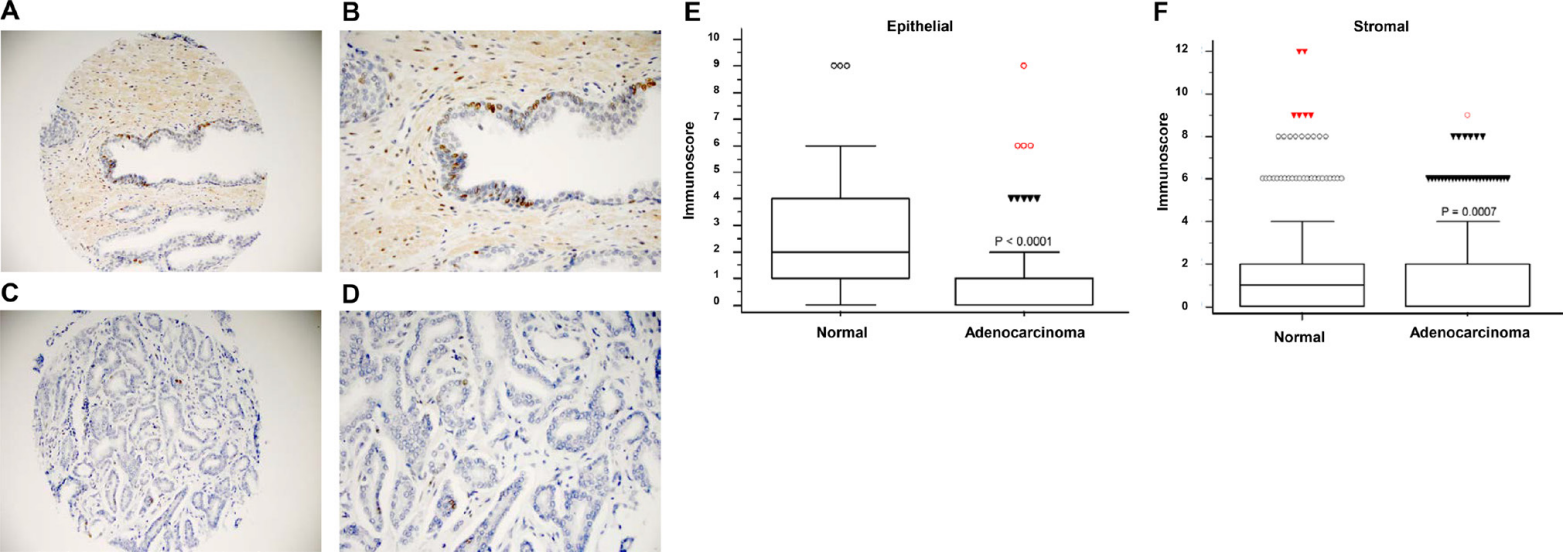
*HIST1H1A* expression is down-regulated in prostate adenocarcinoma Representative images of histological sections showing positive staining of H1.1 in normal prostate at 20× and 40× (**A** and **B**), and prostate adenocarcinoma at 20× and 40× (**C** and **D**). Box plots representing immunoscore (immunointensity × percentage score) in prostate epithelial tissue, *P* = 1.0 × 10^-4^ (**E**), and prostate stromal tissue, *P* = 7.0 × 10^-4^ (**F**). *P*-values were determined using Wilcoxon rank sum test (*n* = 80 cases vs. *n* = 80 control).

To investigate the clinical relevance of changes in H1.1 expression in PC, we performed immunohistochemistry (IHC) staining using prostate tissue microarray (TMA). TMAs, which were obtained from The Prostate Cancer Biorepository Network, consisted of 80 cases of normal prostate epithelial and matched adenocarcinoma samples. Clinical characteristics of patient samples can be found in Supplementary Table 3. Strong H1.1 staining was observed in normal prostate epithelium (Figure 2A and 2B) compared to weaker staining in the prostate adenocarcinoma (Figure 2C and 2D). Significantly higher immunoscores (intensity of positive staining × percentage of positive cells) of H1.1 staining was observed in normal prostate epithelial tissue (*P* < 1.0 × 10^–4^; Figure 2E) as well as stromal tissue (*P* = 7.0 × 10^–4^; Figure 2F) compared to adenocarcinoma tissue.

### *HIST1H1A* suppresses growth and metastasis in the androgen receptor negative PC-3 cells

**Figure 3:**
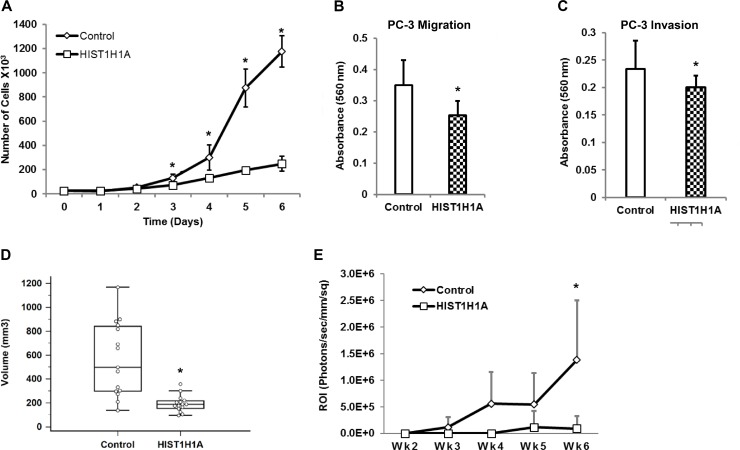
*HIST1H1A* inhibits cell growth, migration, and invasion both *in vitro* and *in vivo* in androgen receptor negative PC-3 cells Growth was monitored in three clonal isolates of PC-3 cells over-expressing *HIST1H1A* or control vector, *P* (^*^) < 0.01 (**A**). *In vitro* cell migration was determined in PC-3 cells over-expressing *HIST1H1A* or control vector by monitoring migration of cells across trans-well membrane coated with collagen, *P* (^*^) = 0.002 (**B**). *In vitro* cell invasion was determined in PC-3 cell over-expressing *HIST1H1A* or control vector by monitoring the ability of cells to invade a Matrigel matrix, *P* (^*^) = 0.042 (**C**). Cells expressing *HIST1H1A* or control vector were injected into the flanks of NU/J mice, and tumor size was measured once a week for 5 weeks using a digital caliper. Results are expressed as tumor volume, Volume = (Width^2^ × Length)/2, (*n* = 15), *P* (^*^) = 6.79 × 10^-5^ (**D**). PC-3 Luc cells over-expressing *HIST1H1A* or control vector and co-expressing the luciferase gene were injected into the left ventricle of NU/J male mice. Bioluminescence was quantified by imaging mice weekly using an IVIS Xenogen chamber to monitor dissemination of cancer cells, which is measured by photon flux (P/sec/mm/sq) (*n* = 12), *P* (^*^) = 0.001 (**E**). Results are presented as mean + SD of at least two experiments, statistical significance was calculated using the Student’s *T*-test, or ANOVA with *P* (^*^) < 0.05 representing statistical significance between *HIST1H1A* and control vector group.

To better understand the role *HIST1H1A* plays in PC aggressiveness, we stably over- expressed *HIST1H1A* in the aggressive AR-negative human PC cell line PC-3 using lentiviral transduction. Control cells were generated by transducing PC-3 cells with lentivirus containing an empty vector. *HIST1H1A* expression was confirmed using RT-qPCR and Western blot (Supplementary Figure 1A and 1B). To determine how *in vitro* growth rates were affected in cells expressing *HIST1H1A* versus control, we performed growth curve analysis. Over-expression of *HIST1H1A* significantly suppressed cell growth on day six compared to control, *P* = 2.08 × 10^–8^ (Figure 3A). To explore *HIST1H1A* involvement in cell migration and invasion, we employed a trans-well migration system, which allow movement of cells across a membrane coated with collagen IV or Matrigel, respectively. Over-expression of *HIST1H1A* significantly suppressed migration (average absorbance 560 nm = 0.25 ± 0.05) versus control (average absorbance 560 nm = 0.35 ± 0.08, *P* = 0.002), and invasion (average absorbance 560 nm = 0.20 ± 0.02) versus control (average absorbance 560 nm = 0.23 ± 0.05, *P* = 0.04; Figure 3B and 3C).

Subsequently, we examined the effect of *HIST1H1A* over-expression on tumor growth and dissemination *in vivo*. We investigated the ability of *HIST1H1A* to modulate tumor growth in a xenograft flank assay by injecting control cells, or cells over-expressing *HIST1H1A* into the flanks of NU/J mice and observed tumor growth over a five-week period. *HIST1H1A* significantly suppressed tumor volume compared to the control group (average tumor volume = 212 ± 133 mm^3^ versus 1,305 ± 896 mm^3^, *P* = 6.79 × 10^–5^; Figure 3D). To evaluate the effect of *HIST1H1A* on tumor dissemination *in vivo*, we performed intra-cardiac injections in NU/J mice using PC-3 cells tagged with luciferase (PC-3 Luc), and over-expressing either *HIST1H1A* or control vector. Tumor dissemination was determined by quantifying bioluminescent signals of cells over-expressing either *HIST1H1A* or control vector over six weeks. A significant reduction in dissemination of PC-3 cells was observed in the *HIST1H1A* group compared to the control group (average flux 9.17 × 10^4^ ± 2.34 × 10^5^ versus 1.38 × 10^6^ ± 1.12 × 10^6^, *P* = 0.001; Figure 3E).

### *HIST1H1A* increases the aggressiveness of the androgen receptor-positive human prostate cancer LNCaP cell line

**Figure 4:**
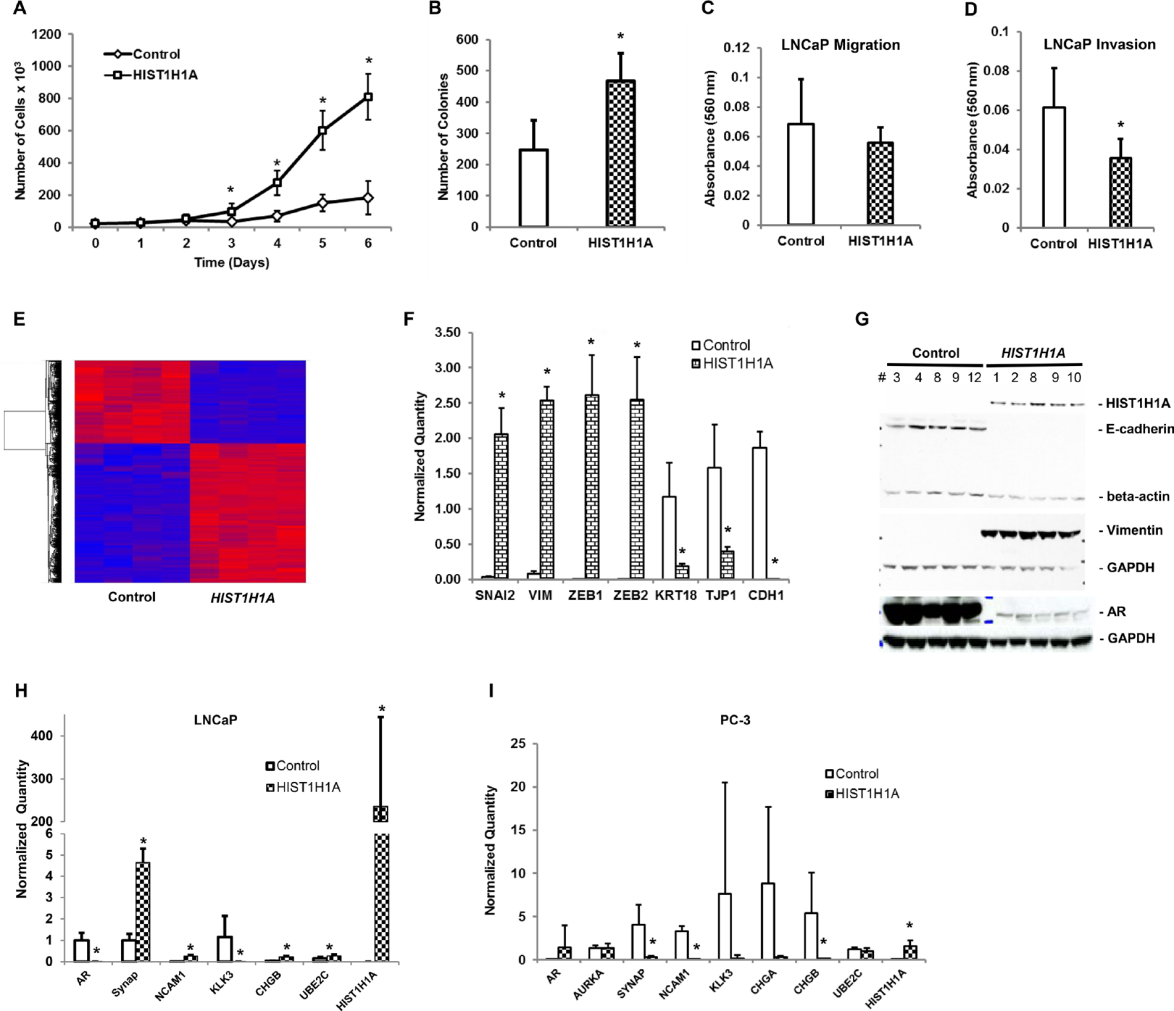
*HIST1H1A* enhanced the expression of neuroendocrine and epithelial-to-mesenchymal markers in the androgen receptor-positive LNCaP human prostate tumor cells Growth was monitored in three clonal isolates of LNCaP cells over-expressing *HIST1H1A* or control vector, *P* (^*^) < 0.001 (**A**). Growth in suspension was monitored in LNCaP cells over-expressing *HIST1H1A* or control vector by growing cells in soft agar, *P* (^*^) = 0.014 (**B**). *In vitro* cell migration was determined in LNCaP cells over-expressing *HIST1H1A* or control vector by monitoring migration of cells across trans-well membrane coated with collagen, *P* = n.s. (**C**). *In vitro* cell invasion was determined in LNCaP cells over-expressing *HIST1H1A* or control vector by monitoring the ability of cells to invade a Matrigel matrix, *P* (^*^) = 6.22 × 10^-4^ (**D**). Global gene expression was quantified in LNCaP cells over-expressing *HIST1H1A* or control vector using microarray analysis. Heat map represents relative gene expression of four clonal isolates over-expressing *HIST1H1A* or control vector (**E**). qRT-PCR was used to quantify gene expression of EMT markers in four clonal isolates of LNCaP cells over-expressing *HIST1H1A* or control vector, *P* (^*^) < 0.05 (**F**). Western blot was used to quantify protein expression of EMT markers in LNCaP cells over-expressing *HIST1H1A* or control vector, GAPDH served as a loading control (**G**). qRT-PCR was used to quantify gene expression of NE markers in four clonal isolates of LNCaP cells, *P* (^*^) < 0.05 (**H**) and PC-3 cells, *P* (^*^) < 0.05 (**I**). Results are presented as mean + SD of three experiments, statistical significance was calculated using the Student’s *T*-test with *P* (^*^) < 0.05 representing statistical significance between *HIST1H1A* and control vector group.

To characterize the functional role *HIST1H1A* plays in an androgen receptor positive environment, we used lentiviral transduction techniques to stably over-express *HIST1H1A* or a control vector in the LNCaP PC cell line as was previously performed in PC-3 cells. Growth curve analysis revealed that cells over-expressing *HIST1H1A* exhibited a significant increase in cell growth compared to the control group (*P* = 7.45 × 10^–15^; Figure 4A). We next performed soft agar assay to determine the effect of *HIST1H1A* over-expression on cell growth in 3D culture. Over-expression of *HIST1H1A* significantly enhanced the number of colonies found growing in suspension compared to the control group (468 ± 87.5 versus 247 ± 96 colonies, *P* = 0.014; Figure 4B). To investigate the *in vitro* migratory and invasive potential of cells expressing *HIST1H1A* in an AR-positive environment, we performed trans-well assays. LNCaP cell migration was not significantly impacted by *HIST1H1A* over-expression (Figure 4C). However, invasion was significantly decreased with the over-expression of *HIST1H1A* compared to the control group (average absorbance 560 nm = 0.030 ± 0.009) versus (average absorbance 560 nm = 0.060 ± 0.020; *P* = 6.22 × 10^–4^; Figure 4D). Table 1 presents a summary of *in vitro* and *in vivo* studies performed in PC-3 and LNCaP cells over-expressing *HIST1H1A* relative to cells expressing the control vector.

**Table 1:** Summary of *in vitro* and *in vivo* studies using PC-3 and LNCaP cell lines over-expressing *HIST1H1A*

*In Vitro* and *In Vivo* assays	PC-3 cells	LNCaP cells
Soft agar assay	N/A	Increase
Growth assay	Decrease	Increase
Migration	Decrease	n.s Decrease
Invasion	Decrease	Decrease
Flank assay	Decrease	N/A
Intra-cardiac injections	Decrease	N/A

n.s represent non-significant.

Data represent results compared to cells expressing the control vector in each cell lines.

### Over-expression of *HIST1H1A* impacts global gene expression in prostate cancer cell lines

To gain insight into the mechanism underlying the influence of *HIST1H1A* in promoting a more aggressive PC phenotype in LNCaP cells, we used microarray analysis to evaluate the expression profile of clonal isolates expressing either *HIST1H1A* or control vector. Over 1,900 transcripts were found to be significantly dysregulated in response to *HIST1H1A* over-expression (fold change > ±2; false discovery rate [FDR] < 0.050; Figure 4E, Supplementary Table 4). IPA identified several canonical signaling pathways and molecules affected by *HIST1H1A* over-expression, including PTEN signaling (FDR = 1.55 × 10^–4^), regulation of EMT (FDR = 3.98 × 10^–4^), and the WNT/Ca^+^ pathway (FDR = 8.51 × 10^–4^) (Supplementary Tables 5 and 6).

Since *HIST1H1A* over-expression affected several factors of the EMT signaling pathway, we sought to validate several genes found in the EMT pathway by performing qRT-PCR. Our analyses confirmed that *HIST1H1A* over-expression in LNCaP cells significantly affected several prominent EMT markers. Gene expression for mesenchymal marker Vimentin (*VIM)*, and transcription factors Slug (*SNAI2*), Zinc finger E-box binding homeobox (*ZEB)* 1 and 2 were significantly upregulated; *VIM* (fold change = 34.32 ± 2.63, *P* = 4.29 × 10^–6^), *SNAI2* (fold change = 63.91 ± 11.61, *P* = 2.63 × 10^–4^), *ZEB1* (fold change = 2.07 × 10^3^ ± 4.47 × 10^2^, *P* = 4.93 × 10^–4^) and *ZEB2* (fold change = 4.23 × 10^3^ ± 9.90 × 10^2^, *P* = 6.74 × 10^–4^). However, gene expression for epithelial markers Keratin 18 (*KRT18*), Tight junction protein 1 (*TJP1*) and E-cadherin (*CDH1*) were significantly suppressed; *KRT18* (fold change = 1.60 × 10^–1^ ± 2.91 × 10^–2^, *P* = 0.001), *TJP1* (fold change = 2.5 × 10^–1^ ± 4.37 × 10^–2^, *P* = 0.01), and *CDH1* (fold change = 5.55 × 10^–4^ ± 4.97 × 10^–4^, *P* = 5.72 × 10^–5^; Figure 4F). Western blot analysis confirmed that over-expression of *HIST1H1A* affects the protein expression of several of these EMT molecules. In particular, loss of protein expression of epithelial marker E-cadherin in the presence of *HIST1H1A*, was associated with increase protein expression of Vimentin (Figure 4G, Supplementary Figure 2). Taken together, these data suggest that *HIST1H1A* promotes aggressive PC development. Furthermore, aggressive PC development occurs simultaneously with aberrant changes in EMT factors at the gene and protein level.

Among the list of dysregulated transcripts identified in LNCaP cells using microarray analysis, there were several NE genes that are associated with aggressive PC (Table 2, Supplementary Table 4). This is of interest given that *Hist1h1a* was identified as an aggressive disease modifier using the TRAMP mouse model of NEPC [15]. To validate the microarray results, we performed qRT-PCR analysis, which confirmed that over-expression of *HIST1H1A* significantly enhanced gene expression of Synaptophysin (*SYN*) (fold change = 4.64 ± 0.66, *P* = 3.55 × 10^–6^), *Neural Cell Adhesion Molecule 1* (*NCAM1*) (fold change = 1.06 × 10^3^ ± 1.47 × 10^2^, *P* = 22.02 × 10^–5^), *Chromogranin B* (*CHGB*) (fold change = 6.7 ± 1.46, *P* = 3.91 × 10^–5^), and *Ubiquitin Conjugating Enzyme E2 C* (*UBE2C*) (fold change = 1.70 ± 0.45, *P* = 0.04), but significantly suppressed *AR* (fold change = 3.78 × 10^–4^ ± 1.29 × 10^–4^, *P* = 1.99 × 10^–4^) and *Kallikrein-Related Pepidase 3* (*KLK3*) (fold change = 3.73 × 10^–6^ ± 5.77 × 10^–6^, *P* = 0.03) (Figure 4H). Conversely, over-expression of *HIST1H1A* in the aggressive PC-3 cell line had an opposite effect on NE markers. Specifically, qRT-PCR analysis demonstrated that *HIST1H1A* over-expression in PC-3 cells significantly suppressed the expression of *SYN* (fold change = 8.00 × 10^–2^ ± 0.03, *P* = 0.008), *NCAM1* (fold change = 5.51 × 10^–5^ ± 5.13 × 10^–5^, *P* = 4.03 × 10^–6^) and *CHGB* (fold change = 2.56 × 10^–2^ ± 7.67 × 10^–3^, *P* = 0.04; Figure 4I) compared to the control group.

**Table 2:** Microarray data analysis of neuroendocrine gene transcripts dysregulated in LNCaP cells over-expressing *HIST1H1A*

Transcript ID	Gene name	Gene symbol	RefSeq	*P*-Value (*HIST1H1A* vs. Control)	Fold-change (*HIST1H1A* vs. Control)
17104313	Androgen receptor	*AR*	NM_000044	0.00059	–13.268
16920548	Aurora kinase A	*AURKA*	NM_003600	0.97544	1.00369
16787650	Chromogranin A	*CHGA*	NM_001275	0.64767	–1.05805
16911201	Chromogranin B	*CHGB*	NM_001819	0.76382	1.03232
16864616	Kallikrein-related Peptidase 3	*KLK3*	NM_001030047	0.00069	–12.0945
16731297	Neural cell adhesion Molecule 1	NCAM1	NM_001076682	0.00129	2.43506
17110835	Synaptophysin	SYP	NM_003179	0.18923	1.1505
16914315	Ubiquitin-conjugating enzyme E2C	UBE2C	NM_001281741	0.20479	1.22344

### ATAC-seq analysis identified enhanced open chromatin regions in LNCaP cells over-expressing *HIST1H1A*

**Figure 5:**
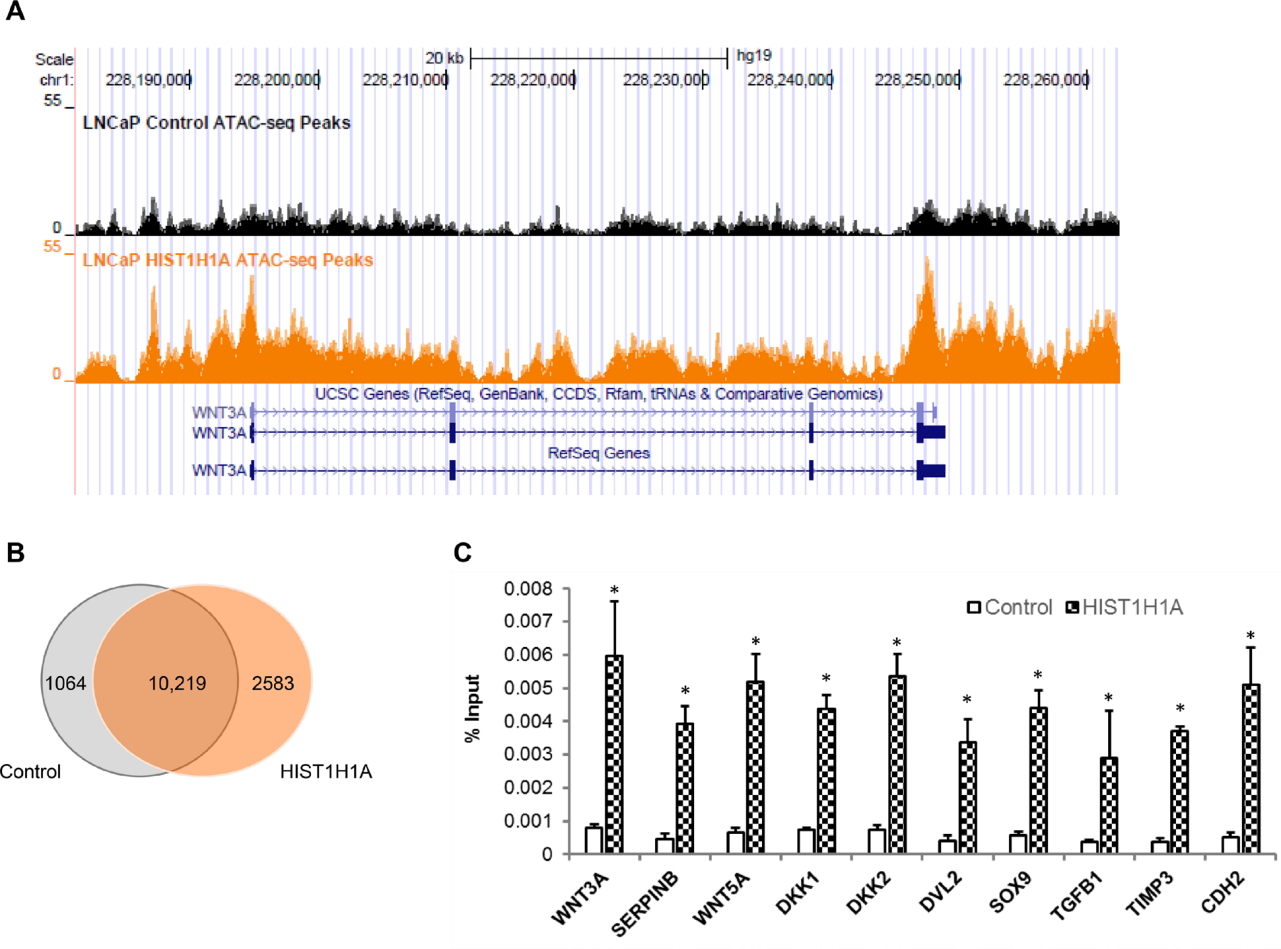
ATAC-seq analysis identified enhanced open chromatin regions in the presence of *HIST1H1A* Tracks from UCSC genome browser following ATAC-sequencing was generated from LNCaP cells over-expressing either *HIST1H1A* or control vector. Analysis was done in triplicate and peak regions are representative of one sample; control peaks are shown in black and *HIST1H1A* peaks are shown in orange (**A**). Venn diagram showing overlap in genes found in open chromatin region following ATAC-seq analysis of LNCaP cells over-expressing *HIST1H1A* and control vector (**B**). Genes identified in the ATAC-seq analysis were validated using ChIP-qPCR in LNCaP cells over-expressing *HIST1H1A* or control vector. Graphs represent the average of three clonal isolates, presented as mean + SD, *P* (^*^) < 0.05 (**C**). Statistical significance was calculated using the Student’s *T*-test with *P* (^*^) < 0.05 representing statistical significance between *HIST1H1A* and control vector group.

To investigate how *HIST1H1A* influences chromatin compaction, we used ATAC-seq, which is based on the integration of Tn5 transposase in the open chromatin region [26]. Three LNCaP cell clonal isolates stably over-expressing either *HIST1H1A* or control vector were used to generate sequencing libraries. Figure 5A represent regions of both increased and decreased chromatin compaction in *HIST1H1A* versus the control group, as reflected by varying degrees of peak regions. ATAC-seq analysis indicated that over-expression of *HIST1H1A* increased the number of open chromatin regions to 19,277 compared with 16,173 in control cells. There was a significant overlap of genes harboring open chromatin regions, with 10,219 (74%) of these genes demonstrating overlap between *HIST1H1A* and control group (Figure 5B). However, there were also unique subsets of genes that lost open chromatin regions (1,064; 7.6%), and gained open chromatin regions (2,583; 18.6%) with over-expression of HIST1H1A (Figure 5B). A complete list of genes identified in the *HIST1H1A* and control groups can be found in Supplementary Tables 7 and 8 respectively. To determine the pathways affected by these changes in chromatin landscape attributed to *HIST1H1A* over-expression, IPA was performed using the list of genes identified as unique to either the *HIST1H1A* or control group. Three distinct clusters were identified based on their functional characteristics (Supplementary Tables 9–11). Two lists were associated with LNCaP cells over-expressing *HIST1H1A*, one which consist of WNT3a target molecules, and the second consist of WNT signaling molecules. The third list include molecules involve in androgen biosynthesis and was associated with LNCaP cells over-expressing the control vector.

To confirm that *HIST1H1A* is involved in regulating the expression of WNT pathway target genes, we performed ChIP-qPCR to investigate protein-DNA interactions at specific genomic sites identified from the ATAC-seq analysis. Fragments of cross-linked chromatin of hemagglutinin (HA)-tagged *HIST1H1A* were immunoprecipitated from three LNCaP cell clonal isolates over-expressing *HIST1H1A* or control vector using an HA antibody. Quantitative-PCR was performed using several sets of primers targeting molecules involved in both the canonical and non-canonical WNT pathways. Immunoglobulin G (IgG) was used as a negative control. In samples over expressing *HIST1H1A*, signals were enriched for *WNT3A*, *SERPINB*, *WNT5A*, *DKK1*, *DKK2*, *DVL2*, *SOX9*, *TGFβ1*, *TIMP3*, and *CDH2* compared to control group, *P* (^*^) < 0.05 (Figure 5C). These data suggest that in an AR-positive environment, *HIST1H1A* may be involved in promoting a NE phenotype by influencing members of the WNT pathway.

## DISCUSSION

In this study, we explored the functional role *HIST1H1A* plays in the development of aggressive PC. We hypothesized that *HIST1H1A* modulates chromatin structure, which in turn influences the expression of genes and pathways critical to the development of aggressive PC.

An earlier study demonstrated that mice lacking *Hist1h1a* exhibit normal development [27]. While the functional role of *HIST1H1A* in PC has not been previously investigated, there are some studies investigating the expression of *HIST1H1A* and other linker histones genes in different cancer types. The expression of *HIST1H1A* along with *HIST1H1E*, *HIF0,* and *HIFX* were significantly reduced in ovarian malignant adenocarcinoma compared to benign tumor, while the linker histone *HIST1H1D* showed the reverse trend [28]. In addition, hierarchical clustering of gene expression patterns further indicate that these four linker histones can discriminate between ovarian adenomas and adenocarcinoma, suggesting their potential as biomarkers for aggressive disease [28]. In an immuno-histochemistry study, *HIST1H1B* was shown to be positively correlated with tumor grade, in that its nuclear expression increased with the grade of pulmonary NE carcinomas [29]. *HIST1H1B* protein expression was also assessed in human prostate adenocarcinoma, which revealed strong nuclear reactivity in most prostate adenocarcinoma cases compared to the benign prostatic glands [30]. In addition, *HIST1H1B* reactivity was more positively associated with Gleason Score 4 and 5, pointing to the potential of this linker histone as a diagnostic tool [30].

Our study indicates for the first time that *HIST1H1A* expression is significantly suppressed in human prostate adenocarcinoma compared to its normal counterpart using prostate TMA. Whereby, *in silico* validation using the GSE21032 cohorts suggested that *HIST1H1A* expression was associated with Gleason Score, we found no significant association between Gleason Score and H1.1 TMA staining. However, the TMA data presented here was somewhat underpowered (*n* = 80 cases vs. *n* = 80 controls), and a larger-scale analysis is required to address an association of H1.1 levels with tumor grade. Additionally, it would be of interest to determine whether H1.1 expression differs between prostate adenocarcinomas and NEPC. The latter will be the emphasis of future studies.

In our study, *HIST1H1A* over-expression in LNCaP cells significantly suppressed AR expression at the gene and protein level. In addition, opposing effects were observed with regards to the expression of a panel of NE marker genes in each of the cell lines. *HIST1H1A* expression enhances NE marker expression in LNCaP cells, yet suppressing their expression in PC-3 cells. These differences in the two cell lines induced by *HIST1H1A* over-expression may account for the differences in cell growth in LNCaP and PC-3 cells as well as tumor growth and metastasis in PC-3 cells, and supports a possible role for *HIST1H1A* in regulating AR signaling and EMT. The mechanisms through which *HIST1H1A* exert its effects in AR signaling and EMT are presently unclear, and is the focus of ongoing studies.

It was previously reported that activation of the WNT signaling pathway through β-catenin in LNCaP cells induced the expression of multiple NE markers [31]. In addition, histological studies of mouse prostate tissue following activation of WNT signaling through β-catenin showed evidence of elevated levels of chromogranin A, as well as the forkhead transcription factor, FOXA2, which are factors associated with neuroendocrine differentiation. Several studies have indicated that there is a correlation between loss of AR and NE differentiation [32–34]. In our study, both Western blot analysis and gene expression demonstrated that *HIST1H1A* over-expression in LNCaP cells lead to down-regulation of AR. In addition, pathway analysis identified WNT/β-catenin signaling as the top canonical pathway associated with *HIST1H1A* over-expression. The WNT signaling pathway is involved in both embryogenesis and tumorigenesis [35]. In the prostatic epithelial tissue, WNT signaling regulates cell proliferation, differentiation, and maintenance through its interaction with β-catenin [36–38]. Mechanistically, the interaction between WNT signaling and AR signaling differs based on PC stage: WNT/ β-catenin signaling is associated with AR-target gene transcription in hormone naïve PC cells; however, in castrate-resistant PC, both AR and WNT/ β-catenin signaling stimulate each other resulting in activation of genes involved in PC cell growth in an androgen-independent manner [39]. In addition, WNT signaling was identified as the most androgen regulated pathway, during early prostate development [40]. In particular, introduction of AR mutation into the prostate epithelia of TRAMP mice resulted in enhanced tumor formation and growth as a consequence of stimulation of the non-canonical WNT signaling pathway, particularly through its ligand, WNT-5A [41].

In the presence of WNT signaling, phosphorylation of β-catenin is inhibited, allowing its translocation to the nucleus where it binds to transcription factors of the TCF/LEF family and promote processes such as EMT [35]. EMT is a signaling pathway invoked during various stages of embryogenesis, including gastrulation, neural tube formation, as well as non-developmental processes such as wound healing. In addition, EMT is well documented in cancer progression and metastasis [35, 38, 42]. Cells that are locally invasive have been shown to lose their adherent characteristics through reduction of cell adhesion molecules such as E-Cadherin, and up-regulation of proteins such as Vimentin, and N-Cadherin. Several transcription factors such as Snail1 and Snail2 (Slug), Zeb1, Zeb2, and Twist1 have all been implicated in regulation of these cell adhesion molecules [35, 38, 42, 43]. In the current study, we demonstrated that *HIST1H1A* modulates the expression of several of these genes involved in EMT.

In summary, this study suggests a plausible mechanism underlying the effect of *HIST1H1A* in aggressive human prostate tumorigenesis. As ADT treatment increases, it is expected that the proportion of patients suffering from the aggressive NEPC sub-type will also increase. Therefore, a clearer understanding of the mechanisms that underlie aggressive PC development will assist researchers in the development of better treatment options. Overall, we have provided evidence that systems genetics can be used to show how hereditary variation influences aggressive PC susceptibility.

## MATERIALS AND METHODS

### Generation of stable cell line expressing *HIST1H1A*

Human prostate tumor cell line LNCaP was obtained from American Type Culture Collection (ATCC), and grown in RPMI cell culture media. PC-3 Luc cell line was modified from PC-3 human prostate tumor cells to express luciferase and were donated from Dr. Kathleen Kelly at NCI/NIH [44]. PC-3 Luc cells were grown in DMEM cell culture media. Both LNCaP and PC-3 Luc cell growth media were supplemented with 10% FBS and 1% Penicillin/Streptomycin, and cell cultures were maintained at 37° C and 5% CO_2_. *HIST1H1A* (GE Dharmacon, Lafayette, CO, USA, cat # OHS6085-213573401) or a control vector were stably expressed in both cell lines using lentiviral transduction as previously described [45]. Following transduction, LNCaP cells harboring the vector of interest were selected using 3 μg/mL blasticidin, and PC-3 Luc cells were selected using 20 μg/mL blasticidin. Clonal isolates were obtained using serial dilution, and the expression of *HIST1H1A* was confirmed using Western blot and qRT-PCR.

### Cell proliferation and anchorage independent growth assays

For cell proliferation assays, PC-3 Luc and LNCaP clonal isolates stably over-expressing *HIST1H1A* or control vector were seeded at 2.5 × 10^4^ cells per well in 24-well plates and allowed to adhere for 24 hours. For six consecutive days, duplicate wells containing cells were trypsinized and counted in a Cellometer slide counter to determine the growth rate. Anchorage independent growth was assessed using a soft agar colony formation assay, where 1 × 10^3^ cells expressing either *HIST1H1A* or control vector were suspended in a 0.33% agar mixture, and seeded on top of a 0.5% nutrient-agar base in 24-well plates. Each group of cells were plated in duplicate and allowed to grow at 37° C and 5% CO_2_ for two weeks. Cell colonies were stained with 0.005% crystal violet and counted.

### Trans-well cell migration and invasion assay

Cell migration and invasion assay were performed as previously described [16]. Briefly, PC-3 Luc or LNCaP cells stably over-expressing *HIST1H1A* or control vector were seeded at 5 × 10^5^ cells in serum-free media into the upper chamber of 8.0 um 24-well cell inserts (ThermoFisher Scientific). For cell migration assays, membrane of inserts were coated with 5 μg collagen I dissolved in 0.02 M acetic acid. For invasion assays, insert membranes were coated with 30 μg Matrigel (Corning) diluted in 0.01 M Tris (pH 8.0) and 0.7% NaCl. Inserts were placed in 24-well tissue culture dishes containing 10% FBS in cell culture media, which serves as an attractant to the “serum starved” cells within the upper insert. 48 hours later, cells remaining in the upper chamber were gently removed using a cotton swab, and cells still attached to the lower surface (cells that have migrated or invaded across the membrane) were fixed with 4% paraformaldehyde, and stained with crystal violet (0.05% in ethanol). Snapshots of migratory or invading cells were taken, and membranes with attached cells were destained in 300 µL of 2% SDS. Absorbance was read in duplicates at 560 nm using a microplate reader (Molecular Devices, Sunnyvale, CA, USA). Statistical analyses were performed using Student’s *T*-test and data are presented as mean ± SD with *P* < 0.05 considered as significant.

### RNA isolation and gene expression by quantitative real time PCR (qRT-PCR)

Total RNA was extracted from clonal isolates of PC-3 or LNCaP cells expressing *HIST1H1A* or the control vector using RNeasy Plus Mini Kit (QIAGEN). The concentration and purity of isolated RNA was measured using a NanoDrop (Wilmington, DE USA). Total RNA was reversed transcribed using iScript DNA Synthesis Kit (Bio-Rad, Hercules, CA USA) according to the manufacturer’s protocol. qRT-PCR were performed for gene expression using ABI Fast SYBR Green Master Mix (Life Technologies, Grand Island, NY USA) as previously described [45].

### Microarray analysis

Total RNA from LNCaP clonal isolates expressing *HIST1H1A* or control vector were isolated using miRNeasy Mini Kit according to the manufacture’s protocol (QIAGEN, Cat #217004). Samples were processed using Affymetrix Human Gene 2.0 ST Array and GeneChip WT PLUS Reagent Kit (Santa Clara, CA USA) according to the manufacturer’s protocol. Differential expression data was analyzed using Partek Genomic Suite, and heat maps were generated using R as previously described [45]. Omics data was analyzed using Ingenuity Pathway Analysis (IPA) (QIAGEN). For IPA the following parameters were used: All data sources Confidence = experimentally observed; All Species = human; All tissue and primary cells using the stringent filter; Examined both interaction molecules and causal networks. All data from microarray analysis were submitted to Gene Omnibus GSE101982.

### Assay for transposase accessible chromatin (ATAC)-sequencing experiment and analysis

Approximately 5 × 10^4^ cells were taken from a combined pool of three independent clonal isolates of LNCaP cells stably over-expressing *HIST1H1A* or control vector. The cells were then lysed, and the transposition reaction was carried out using the Nextera DNA Sample Prep Kit (Illumina Cat # FC-121-1032). Purification was performed using AmpureXP beads at room temperature. The transposed DNA fragments were amplified using PCR techniques as previously described (Cycles: 1–72° C, 5 mins; 2–98° C, 30 secs; 3–98° C, 10 secs; 4–63° C, 30 secs; 5–72° C, 1 min; 6-Repeat steps 3–5, 4×; 7-Hold at 4° C) [26]. ATAC-seq data was generated on the Illumina HiSeq2500 platform. Each sample was sequenced on four separate lanes, and single-end ATAC-seq data were obtained. ATAC-seq reads that passed the Illumina platform quality check were used for downstream analyses. ATAC-seq reads were mapped to the human hg19 reference genome sequence using the BWA aligner (BWA mem; v. 0.7.12). Unambiguously mapped reads were selected using samtools view with option -q 1. Mapping data for corresponding samples were merged using samtools merge, and bamToBed (v. 2.10.0) was used to generate BED files containing the mapped positions for the ATAC-seq data. ATAC-seq reads that mapped to genomic regions of low mappability (centromere, telomere, and satellite repeats) were removed. PCR duplicates were also removed by selecting only one read that mapped to a genomic position in the same orientation. ATAC-seq peak calling was performed with the MACS2 software (v. 2.1.1) using the callpeak command with the following options: —nomodel —shift 100 —extsize 200. Following ATAC-sequencing analysis, IPA analysis was performed. For IPA the following parameters were used: All data sources Confidence = experimentally observed; All Species = human; All tissue and primary cells using the stringent filter; Examined both interaction molecules and causal networks.

### Chromatin immunoprecipitation (ChIP) and validation using qRT-PCR

ChIP-qPCR was used to validate target genes derived from the ATAC-seq analysis. Three independent clonal isolates from LNCaP cells stably over-expressing *HIST1H1A* or control vector were used for ChIP assays as previously described [45]. Briefly, 1% formaldehyde was used to fixed cells, followed by cell lysis. Cell lysates were pre-cleared with Protein G Sepharose beads (GE Healthcare), then incubated with *HIST1H1A* (HPA043753, Sigma Life Science) or IgG (12-370, Millipore), and protein G Sepharose beads were added for overnight incubation at 4° C. NaCl was used for reverse cross-linking, and DNA was extracted using Qiaquick PCR purification kit (Qiagen). The DNA product was used for ChIP-qPCR analysis, and samples were amplified in duplicates using ABI Fast SYBR Green Master Mix (Life Technologies, Grand Island, NY USA). Student’s *T*-tests was used to calculate statistical significance, and data are presented as mean ± SD with *P* < 0.05 being considered significant.

### Western blot

Protein expression in LNCaP and PC-3 cells was determined by Western blotting. Protein extraction was carried out using chromatin extraction buffer containing Complete Protease inhibitor cocktail (Roche, Germany). Protein concentration in the supernatant isolated from LNCaP cells was determined using a standard protocol of the Bradford assay. 30 μg of protein from each sample was separated using SDS-polyacrylamide gel electrophoresis, then transferred onto PVDF transfer membrane (Millipore) using Trans-BLOT SD Semi-Dry Transfer Cell (BIO-RAD). The membrane was blocked for 1 hour using 5% milk in TBS-T before incubation with the primary antibody (Sigma-Aldrich, St. Louis, MO, USA cat # HPA043753) overnight at 4° C, followed by incubation with secondary antibody (Millipore, Billerica, MA, USA) for 1 hr at room temperature. Immunoblots were developed using enhanced chemiluminescence (Amersham Biosciences, Piscataway, NJ).

### Immunohistochemistry

De-identified human prostate tissue microarray (TMA) samples were obtained from The Prostate Cancer Biorepository Network (PCBN). Frozen paraffin embedded tissue microarrays were dried for 1 hour at 60° C. Deparaffinization, rehydration and epitope retrieval were done using Dako pre-treatment link platform using 50× citrate buffer (pH 6.1). *HIST1H1A* antibody (HPA043753, Sigma Life Science) was diluted 1:500 using Envision Flex Antibody Diluent (Dako). Automated IHC with Autostainer Link 48 (Dako) was performed using Envision Flex High-sensitivity visualization system (Dako) kit. Antibody incubation was programmed for 1 hr, and Envision FLEX Rabbit was used to amplify primary antibody signals. Samples were counterstained using EnVision FLEX Hematoxylin (Dako). Sample slides were dehydrated twice for two minutes each in the following solution 70% ethanol, 95% ethanol, 100% ethanol, and xylene, then cover slipped using Permaslip mounting media. Immunostaining of H1.1 in the tissue samples were categorized based on predominant staining intensity in the cells (negative = 0, weak = 1, moderate = 2, and intense = 3); and on the percentage of all positive cells, and weak to strong in the total cell population (negative = 0, 1–5% = 1, 5–25% = 2, 25–50% = 3 and 50–100% = 4). In the cancer cores, only invasive cancers were scored; in the epithelial component, only luminal epithelial cells were scored; and in the stromal components, only myofibroblasts, fibroblasts, and smooth muscle cells were scored. Wilcoxon rank sum test was used to determine statistical significance between prostate normal and adenocarcinoma group with *P* < 0.05 representing significance.

### *In vivo* tumor xenograft and metastasis assay

To monitor tumor growth that might be influenced by over-expression of *HIST1H1A*, 1 × 10^6^ PC-3 Luc cells over-expressing *HIST1H1A* or control vector were re-suspended in 50 μL phosphate buffered saline (PBS) and 50 μL Matrigel (Corning, Bedford MA). Cells were injected subcutaneously into the flanks of 15 six-week old NU/J male mice (Jackson Laboratory, Bar Harbor ME). Tumor growth was measured once a week for six weeks using a digital caliper. Volume of the tumors was calculated using the formula: Volume = (Width^2^ × Length)/2. The results are presented as mean ± SD.

The ability of cells to disseminate to distant sites *in vivo* was assessed using the intra-cardiac metastasis assay as previously described [17]. Briefly, 12 six-week old male NU/J mice were injected with 1 × 10^5^ PC-3-Luc cells over-expressing either *HIST1H1A* or control vector into the left cardiac ventricle. To monitor dissemination of tumor cells, mice were anesthetized with isoflurane, and injected with D-luciferin (150 mg/kg body weight). Bioluminescent images of tumors developing in the mice were acquired using the *in vivo* Xtreme Imager (Bruker, Billerica MA). The experiment was terminated when mice weight dropped by 10% of initial body weight or six weeks post-intracardiac injection. At termination of the experiment, necropsies were performed and mice exhibiting tumor growth in the chest cavity were excluded from data analysis due to cell spillage at the time of injection. Results are represented as mean + SD. Both *in vivo* assays were performed at least twice. In the tumor xenograft experiment the Student’s *T*-test was used to determine significance and for the metastasis experiment ANOVA was used to determine significance, with *P* < 0.05 representing significance. All experiments utilizing mice were approved and performed in compliance with the National Human Genome Research Institute Animal Care and Use Committee’s guidelines.

### Gene analysis in human expression datasets

Logistic regression analysis was performed to determine the association between the expression levels of six *hist1* gene transcripts identified in GWAS analysis with aggressive PC clinical variable, whereby the candidate gene expression level was presented as z-scores, as was previous described [17]. GSE21032 (*N* = 150 PC cases) and GSE46691 (*N* = 545 PC cases) data sets consisting of microarray gene expression data, and the TCGA data set consisting of RNA-seq PC gene expression data was used to determine the z-scores by calculating the SD of the levels of transcript found in each case compared to the mean transcript expression in all tumors. TCGA and GSE21032 consists of tumor gene expression data obtained from cBioPortal for Cancer Genomics [46, 47], and GSE46691 consists of data obtained from Gene Expression Omnibus (https://www.ncbi.nlm.nih.gov/geo/query/acc.cgi?acc=GSE46691). Benjamini-Hochberg FDR for univariate logistic regression *P*-value was performed to correct for multiple testing, where threshold for significance was set as FDR of 5%. Kaplan–Meier survival analysis was performed using Medcalc, where survival time in all cohorts with higher or lower levels of tumor candidate gene expression was compared to all other cases. A z-score of > 2 or <–2 denotes higher or lower levels of gene expression respectively.

### QTL mapping

J/qtl was used to map QTLs in our study as was previously described [15]. Briefly, QTLs were mapped for all traits using a single-QTL analysis, and using a binary model for binary trait, and a non-parametric model for all other traits. Permutation testing was used to test significance levels, using 10,000 permutations. QTL confidence intervals were estimated using 2-LOD support intervals, and QTLs reaching a genome-wide *α* < 0.05 were considered to be of interest.

## SUPPLEMENTARY MATERIALS FIGURES AND TABLES



















## Abbreviations

ADT: androgen deprivation therapy
AR: androgen receptor
ATAC: assay for transposase accessible chromatin
CI: confidence interval
ChIP: Chromatin Immunoprecipitation
Chr: Chromosome
DFS: disease-free survival
EMT: epithelial to mesenchymal transition
FDR: false discovery rate
HA: hemagglutinin
IgG: immunoglobulin G
IHC: immunohistochemistry
IPA: Ingenuity Pathway Analysis
NE: neuroendocrine
NEPC: neuroendocrine prostate cancer
OD: odds ratio
PC: prostate cancer
PSA: prostate specific antigen
PVDF: polyvinylidene difluoride
QTL: quantitative trait locus
TBST: tris-buffered saline-Tween 20)
TMA: tissue microarray
