# Supplementary material for: Prostate cancer susceptibility gene HIST1H1A is a modulator of androgen receptor signaling and epithelial to mesenchymal transition
Source: Oncotarget. 2018 Jun 19;9(47):28532–46. doi: 10.18632/oncotarget.25536 (PMC6033342; doi:10.18632/oncotarget.25536)
Supplement: Supplementary file 1 [file oncotarget-09-28532-s001.pdf]

Prostate cancer susceptibility gene *HIST1H1A* is a modulator of androgen receptor signaling and epithelial to mesenchymal transition

SUPPLEMENTARY MATERIALS

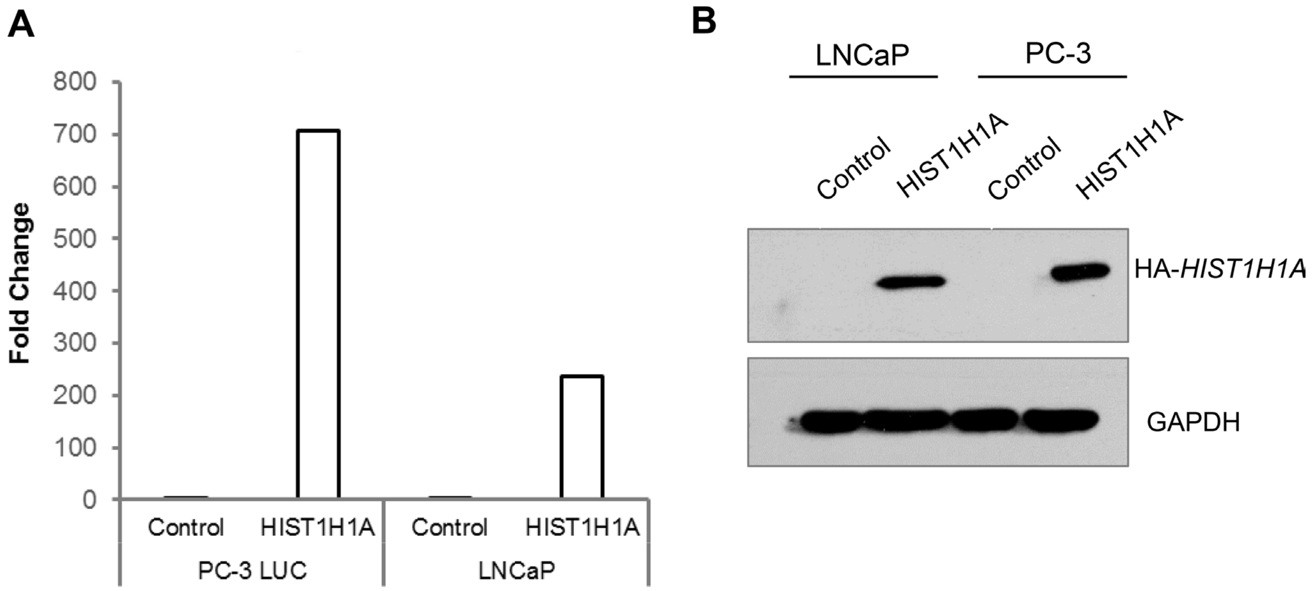

Supplementary Figure 1: Quantitative RT-PCR and Western blot analysis of LNCaP and PC-3 cells. (**A**) qRT-PCR and (**B**) Western blot analysis was used to quantify *HIST1H1A* gene expression and protein expression respectively in clonal isolates of LNCaP and PC-3 cells over-expressing *HIST1H1A* or control vector. HA antibody was used to detect HA-Tagged *HIST1H1A* over-expressed in cell lines, and GAPDH was used as a loading control in Supplementary Figure 1B.

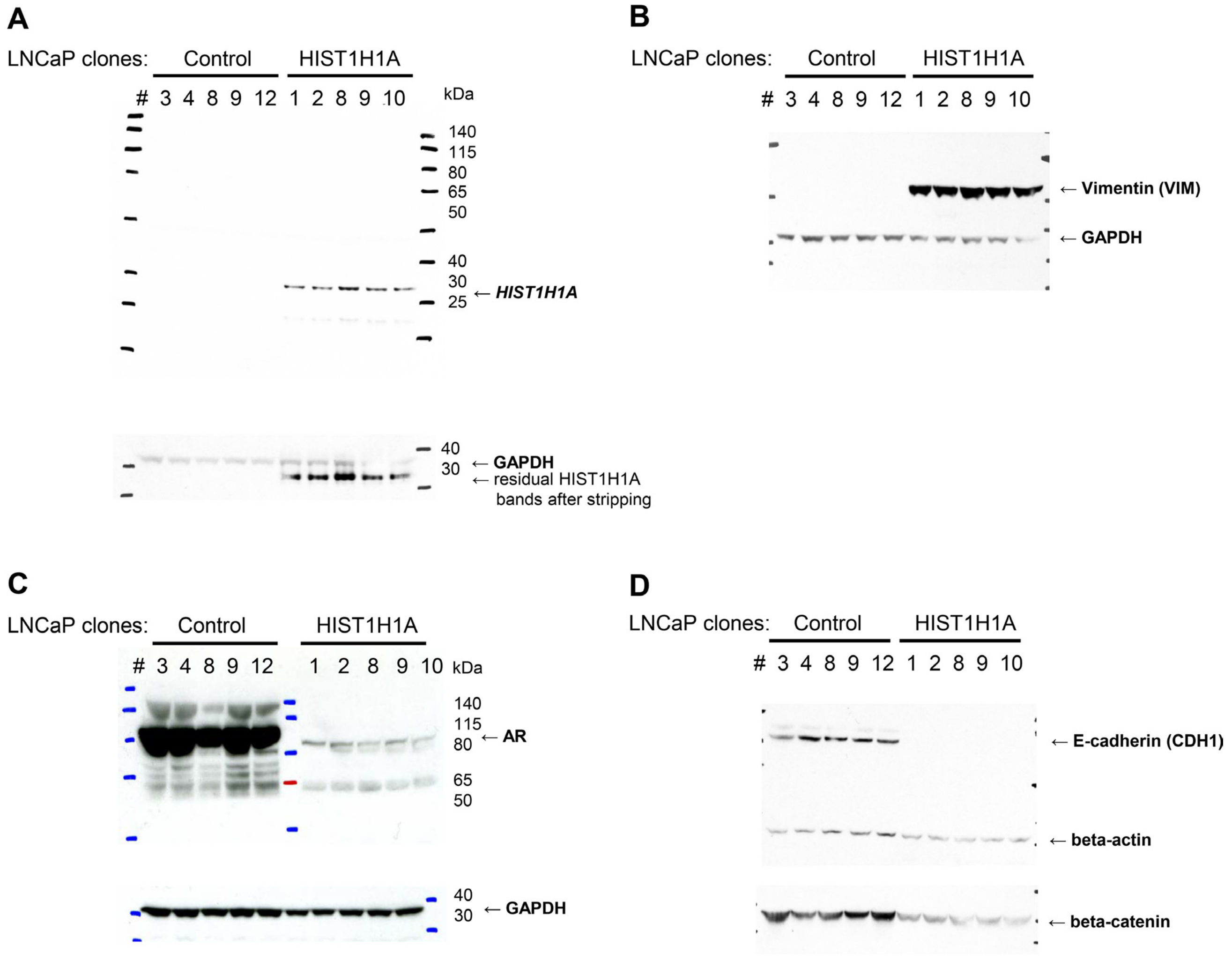

Supplementary Figure 2: Western blot analysis of whole cell lysates from LNCaP cells over expressing *HIST1H1A* or control vector. Protein expression was confirmed for *HIST1H1A* (**A**), Vimentin (**B**), Androgen Receptor (**C**) and E-cadherin and Beta-Catenin (**D**). GAPDH or Beta-Actin was used as a loading control.

Supplementary Table 1: Logistic regression analyses for six genes from the hist1 gene cluster in the GSE46691, GSE21032 and TCGA human prostate cancer gene expression cohort. See Supplementary_Table_1

Supplementary Table 2: Kaplan–Meier Survival analyses for six genes from the hist1 gene cluster in the GSE46691 human prostate cancer gene expression cohort. See Supplementary_Table_2

Supplementary Table 3: Analysis of tissue microarray cores for prostate normal and adenocarcinoma samples. Results indicate the percentage of cells with staining intensity within the epithelial or stromal tissues ranging from negative (0) to 50–100% (4); and the epithelial and stromal intensity score in the core ranging from negative (0) to strong (3). See Supplementary_Table_3

Supplementary Table 4: Microarray data analysis of dysregulated transcripts in LNCaP cell clonal isolates over-expressing *HIST1H1A*. Four clonal isolates from cells expressing either *HIST1H1A* or control vector were prepared for microarray analysis. Results depict fold change in gene expression of cells expressing *HIST1H1A* versus the control vector. See Supplementary_Table_4

Supplementary Table 5: Ingenuity Pathway Analysis identifies several pathways affected by *HIST1H1A* over-expression in LNCaP cells

Ingenuity canonical pathways	FDR	Ratio
Axonal guidance signaling	1.10E-05	1.71E-01
Virus entry via endocytic pathways	1.15E-04	2.70E-01
PTEN signaling	1.55E-04	2.37E-01
Protein Kinase A signaling	3.02E-04	1.61E-01
Regulation of the epithelial-mesenchymal transition pathway	3.98E-04	1.96E-01
Melatonin signaling	5.37E-04	2.71E-01
Gαq signaling	7.24E-04	2.04E-01
Factors promoting cardiogenesis in vertebrates	7.24E-04	2.39E-01
Nitric oxide signaling in the cardiovascular system	8.32E-04	2.30E-01
Wnt/Ca+ pathway	8.51E-04	2.86E-01

Gene expression identified in the microarray analysis (Supplementary Table 4) was used to perform IPA.

Supplementary Table 6: Molecules implicated in the regulation of Epithelial-Mesenchymal Transition that displayed significant fold change with the over-expression of *HIST1H1A* in LNCaP cells

Symbol	Entrez gene name	Entrez gene/gene symbol	Exp *P*-Value	Exp fold change	Type(s)
AKT2	v-akt murine thymoma viral oncogene homolog 2	AKT2	3.03E-04	–1.619	kinase
AKT3	v-akt murine thymoma viral oncogene homolog 3	AKT3	6.82E-04	6.604	kinase
CDH1	cadherin 1	CDH1	4.23E-07	–58.185	other
CDH2	cadherin 2	CDH2	4.22E-06	7.361	other
CLDN3	claudin 3	CLDN3	3.02E-04	–9.46	transmembrane receptor
ESRP2	epithelial splicing regulatory protein 2	ESRP2	8.63E-07	–15.528	other
FGF2	fibroblast growth factor 2 (basic)	FGF2	1.48E-04	3.989	growth factor
FGF5	fibroblast growth factor 5	FGF5	3.22E-04	1.651	growth factor
FGF11	fibroblast growth factor 11	FGF11	1.07E-03	2.777	growth factor
FGFR1	fibroblast growth factor receptor 1	FGFR1	1.20E-04	3.174	kinase
FGFR2	fibroblast growth factor receptor 2	FGFR2	2.59E-04	2.413	kinase
FGFR3	fibroblast growth factor receptor 3	FGFR3	9.62E-04	2.929	kinase
FGFRL1	fibroblast growth factor receptor-like 1	FGFRL1	1.50E-03	–1.85	transmembrane receptor
FZD5	frizzled class receptor 5	FZD5	2.59E-04	–1.519	G-protein coupled receptor
FZD7	frizzled class receptor 7	FZD7	3.06E-05	5.124	G-protein coupled receptor
FZD8	frizzled class receptor 8	FZD8	1.99E-06	14.06	G-protein coupled receptor
JAG2	jagged 2	JAG2	1.28E-03	–2.392	growth factor
JAK1	Janus kinase 1	JAK1	4.00E-07	28.879	kinase
LEF1	lymphoid enhancer binding factor 1	LEF1	1.00E-04	2.701	transcription regulator
LOX	lysyl oxidase	LOX	4.52E-05	2.483	enzyme
MAP2K3	mitogen-activated protein kinase kinase 3	MAP2K3	8.24E-04	–1.765	kinase
MAPK3	mitogen-activated protein kinase 3	MAPK3	2.55E-03	2.487	kinase
MET	MET proto-oncogene, receptor tyrosine kinase	MET	8.67E-07	12.338	kinase
mir-8	microRNA 200c	MIR200C	8.20E-05	–4.609	microRNA
MMP2	matrix metallopeptidase 2	MMP2	6.39E-04	15.238	peptidase
NFKB1	nuclear factor of kappa light polypeptide gene enhancer in B-cells 1	NFKB1	9.75E-04	–1.582	transcription regulator
PIK3CD	phosphatidylinositol-4,5-bisphosphate 3-kinase catalytic subunit delta	PIK3CD	2.03E-03	–2.041	kinase
PIK3R2	phosphoinositide-3-kinase regulatory subunit 2	PIK3R2	1.77E-03	–1.936	kinase
SMO	smoothened, frizzled class receptor	SMO	1.65E-06	5.811	G-protein coupled receptor
SNAI2	snail family zinc finger 2	SNAI2	2.25E-04	16.729	transcription regulator
TCF4	transcription factor 4	TCF4	1.29E-03	2.881	transcription regulator
TCF7L2	transcription factor 7-like 2 (T-cell specific, HMG-box)	TCF7L2	1.60E-05	2.948	transcription regulator
TGFB1	transforming growth factor beta 1	TGFB1	1.60E-03	4.592	growth factor
TGFBR2	transforming growth factor beta receptor II	TGFBR2	1.16E-04	4.883	kinase
WNT5A	wingless-type MMTV integration site family member 5A	WNT5A	2.60E-03	2.301	cytokine
ZEB2	zinc finger E-box binding homeobox 2	ZEB2	9.76E-04	2.067	transcription regulator

IPA identified several molecules affected by *HIST1H1A* over-expression in LNCaP cells.

Supplementary Table 7: ATAC-sequencing data analysis identified transcipts in LNCaP cells over-expressing *HIST1H1A*.
See Supplementary_Table_7

Supplementary Table 8: ATAC-sequencing data analysis identified transcripts in LNCaP cells over-expressing the control vector. See Supplementary_Table_8

Supplementary Table 9: IPA analysis identifies WNT3a target molecules in LNCaP cells over-expressing *HIST1H1A*.
Transcripts identified in the ATAC-sequencing data analysis were used to perform IPA. See Supplementary_Table_9

Supplementary Table 10: IPA analysis identifies WNT signaling molecules in LNCaP cells over-expressing *HIST1H1A*.
Transcripts identified in the ATAC-sequencing data analysis were used to perform IPA. See Supplementary_Table_10

Supplementary Table 11: IPA analysis identifies molecules involved in androgen biosynthesis in LNCaP cells over-expressing the control vector

**Molecules involved in androgen biosynthesis in LNCaP cells expressing control vector**								
**Symbol**	**Entrez gene name**	**Entrez Gene/Gene symbol—human (Hugo/HGNC, entrez gene)**	**Location**	**Type(s)**	**Drug(s)**	**Entrez Gene ID for human**	**Entrez Gene ID for mouse**	**Entrez Gene ID for Rat**
AKR1C3	aldo-keto reductase family 1 member C3	8644	Cytoplasm	enzyme		8644	105349	171516
SRD5A1	steroid 5 alpha-reductase 1	6715	Cytoplasm	enzyme	dutasteride/ tamsulosin, 5-alpha-reductase inhibitor, ethinyl estradiol/ levonorgestrel, levonorgestrel, finasteride, dutasteride	6715	78925	24950
SRD5A2	steroid 5 alpha-reductase 2	6716	Cytoplasm	enzyme	dutasteride/ tamsulosin, azelaic acid, finasteride, dutasteride	6716	94224	64677

Transcripts identified in the ATAC-sequencing data analysis were used to perform IPA.
